# Poly(Butylene Succinate) Hybrid Multi-Walled Carbon Nanotube/Iron Oxide Nanocomposites: Electromagnetic Shielding and Thermal Properties

**DOI:** 10.3390/polym15030515

**Published:** 2023-01-18

**Authors:** Miks Bleija, Oskars Platnieks, Jan Macutkevič, Jūras Banys, Olesja Starkova, Liga Grase, Sergejs Gaidukovs

**Affiliations:** 1 Institute of Polymer Materials, Faculty of Materials Science and Applied Chemistry, Riga Technical University, P. Valdena 3/7, LV-1048 Riga, Latvia; 2 Faculty of Physics, Vilnius University, Sauletekio 9, LT-10222 Vilnius, Lithuania; 3 Institute for Mechanics of Materials, University of Latvia, Jelgavas 3, LV-1004 Riga, Latvia; 4 Institute of Materials and Surface Engineering, Faculty of Materials Science and Applied Chemistry, Riga Technical University, P. Valdena 3/7, LV-1048 Riga, Latvia

**Keywords:** ferrite, electrostatic dissipative, anti-static, electrical conductivity, thermal conductivity, surface resistivity

## Abstract

To address the ever-increasing electromagnetic interference (EMI) pollution, a hybrid filler approach for novel composites was chosen, with a focus on EMI absorbance. Carbon nanofiller loading was limited to 0.6 vol.% in order to create a sustainable and affordable solution. Multiwall carbon nanotubes (MWCNT) and iron oxide (Fe_3_O_4_) nanoparticles were mixed in nine ratios from 0.1 to 0.6 vol.% and 8.0 to 12.0 vol.%, respectively. With the addition of surfactant, excellent particle dispersion was achieved (examined with SEM micrographs) in a bio-based and biodegradable poly(butylene succinate) (PBS) matrix. Hybrid design synergy was assessed for EMI shielding using dielectric spectroscopy in the microwave region and transmittance in the terahertz range. The shielding effectiveness (20–52 dB) was dominated by very high absorption at 30 GHz, while in the 0.1 to 1.0 THz range, transmittance was reduced by up to 6 orders of magnitude. Frequency-independent AC electrical conductivity (from 10^−2^ to 10^7^ Hz) was reached upon adding 0.6 vol.% MWCNT and 10 vol.% Fe_3_O_4_, with a value of around 3.1 × 10^−2^ S/m. Electrical and thermal conductivity were mainly affected by the content of MWCNT filler. The thermal conductivity scaled with the filler content and reached the highest value of 0.309 W/(mK) at 25 °C with the loading of 0.6 vol.% MWCNT and 12 vol.% Fe_3_O_4_. The surface resistivity showed an incremental decrease with an increase in MWCNT loading and was almost unaffected by an increase in iron oxide loading. Thermal conductivity was almost independent of temperature in the measured range of 25 to 45 °C. The nanocomposites serve as biodegradable alternatives to commodity plastic-based materials and are promising in the field of electromagnetic applications, especially for EMI shielding.

## 1. Introduction

With the ever-growing digitalization of our world, we are surrounded by more and more electrical devices. New applications have created a growing field of conductive or semi-conductive polymeric composites. The expected properties of these composites include high wave absorption at the microwave and terahertz frequencies. Carbon nanofillers increase the electrical and thermal conductivity of the polymer matrix [1]. However, to achieve high absorbance, relatively high loadings of carbon nanofillers are needed in the thermoplastic polymer matrix [2]. This limits the sustainability and affordability of EMI shielding materials. To address these challenges, a hybrid filler approach is proposed to keep the carbon nanofiller loading relatively low.

The high aspect ratios of multiwalled carbon nanotubes (MWCNT) allow them to form percolated networks at very low concentrations, and the resulting composites become highly electrically conductive [3]. Iron (II,III) oxide, known as ferrite (Fe_3_O_4_), is a commonly used ferrimagnetic filler. The combination of inorganic carbon and iron nanoparticles enables a hybrid strategy for enhanced electric/magnetic loss in composite materials, which can then be exploited for electromagnetic interference (EMI) shielding properties. The selection of poly(butylene succinate) (PBS) as a matrix for hybrid composites stems from its bio-based origins in combination with its ability to biodegrade in the soil. The mechanical properties of PBS are comparable to commodity plastics such as polyethylene (PE) and polypropylene (PP), in addition to it having conductivity (1.72 × 10^−11^ S/m) several orders higher than other commodity plastics [4].

Traditional EMI shielding materials are usually conductive metals or metal alloys and, as such, function through the reflection of electromagnetic radiation. The interfaces of the fillers and the fillers themselves allow for the EMI to be absorbed by polymer composites and dissipated as heat [5]. By changing the shielding regime from reflection to absorption, the amount of electromagnetic noise in the environment can be decreased. Furthermore, fillers can have synergistic effects on the overall properties of the composite due to interactions not only between the filler and matrix but also between different fillers [6]. Bindu Sharmila et al. reported that the creation of epoxy hybrid reduced graphene oxide/iron oxide composites, where the maximum AC conductivity of 10^−4^ S/m was attained at a frequency of 30 MHz with a filler loading of 5 phr, while the DC conductivity increased up to 10^−6^ S/m, which represented a two- and six-order increase over the neat epoxy, respectively [7].

The literature concerning the application of PBS for EMI shielding, antistatic, and ESD materials is very scarce. Within the field of bio-polyesters, poly(lactic acid) (PLA) and its composites have been investigated more often [8,9,10,11,12,13,14,15]. PBS has been used to create single-filler systems and multi-phase composites with varying degrees of complexity. Lin et al. reported the enhancement of compatibility between PBS and MWCNT by grafting polyetheramines onto the surface of MWCNT [16]. Incorporating 3 wt.% of the modified filler into a PBS matrix decreased the surface resistivity value from 2.35 × 10^14^ to 5.88 × 10^3^ Ω/sq, potentially allowing its use for ESD and EMI shielding purposes. In a study focused on flame-retardant and EMI-shielding polymer composites, Shi et al. combined PBS, thermoplastic polyurethane (TPU), MWCNT, and intumescent flame retardants (IFR) in a segregated double-percolated structure [17]. The composite displayed a very low percolation threshold of 0.22 wt.% MWCNT and an EMI shielding effectiveness of ~45 dB at 8.2 GHz for a 2.5 wt.% filled composite. In another study from the same group, layer-multiplying coextrusion was used to create PBS-MWCNT and TPU-IFR layered composites with EMI shielding efficiency above 30 dB at 8.2–12.4 GHz for 4 wt.% MWCNT filled composites [18]. Luo et al. fabricated a PBS/MWCNT nanocomposite using CO_2_ supercritical foaming for an environmentally friendly EMI shielding material [19]. The foamed composite displayed a percolation threshold of 0.93 wt.% MWCNT and achieved conductivities of 10^−3^ S/m at 4 wt.%. Rincón-Iglesias et al. investigated biodegradable polyester/iron oxide composites with filler loadings from 1 to 10 wt.% [20]. PBS and other polyesters showed very similar performance, with characteristic ferrimagnetic behavior resulting in magnetization with a saturation at around 0.2 T. Saturation values showed good scalability from 0.70 ± 0.1 emu·g^−1^ at 1 wt.% to 7.1 ± 0.1 emu·g^−1^ at 10 wt.% loading.

This research focuses on developing novel composite materials from a biodegradable PBS matrix filled with hybrid fillers (MWCNT and iron oxide). The aim is to combine the electrical and magnetic properties for use in electromagnetic applications primarily focused on EMI shielding. A hybrid system was selected due to the potential synergy in EMI absorption through multiple inner reflections. Composites were prepared using solvent casting, while an ester-based surfactant was used to achieve homogenous nanofiller dispersion. In addition, dielectric spectroscopy in the microwave region, transmittance in the terahertz range, thermal conductivity, broadband AC electrical conductivity, and surface electrical resistivity were investigated.

## 2. Materials and Methods

### 2.1. Materials

All of the materials used in this work are available commercially. Poly(butylene succinate) (PBS) FZ71PM, injection-molding-grade pellets, were purchased from PTT MCC Biochem Co., Ltd., (Bangkok, Thailand). PBS is a semi-crystalline thermoplastic polyester with a density of 1.26 g/cm^3^, MFI 22 g/10 min (190 °C, 2.16 kg); it is entirely biodegradable and partially bio-based (50—85%, DIN certification 8C084). Multiwall carbon nanotubes (MWCNT) NC7000™ (density 1.85 g/cm^3^, L/d 158) were purchased from Nanocyl SA, (Sambreville, Belgium). Iron (II,III) oxide nanoparticles (Fe_3_O_4_) (density 5.1 g/cm^3^, average particle size 100 nm) were purchased from US Research Nanomaterials, Inc., (Houston, TX, USA). Surfactant Ester 80DA (density 1.102 g/cm^3^) is a water- and solvent-soluble synthetic aliphatic ester purchased from ADDAPT Chemicals BV, (Helmond, The Netherlands). Chloroform (CAS No.: 67-66-3) (≥99%) was purchased from Merck KGaA, (Darmstadt, Germany). PBS pellets were dried in a vacuum oven (J.P. Selecta, Barcelona, Spain) before use, according to the manufacturer’s recommendation (70 °C, 24 h). Nanoparticles were used as received without any further purification and were stored in sealed packaging.

### 2.2. Sample Preparation

The nanocomposites were prepared via a solvent casting process. PBS pellets were dissolved in 150 mL of chloroform (concentration 12.5 g/100 mL). At the same time, nanofillers and the surfactant were dispersed in 100 mL of chloroform and then mixed for 45 min using an ultrasonic sonotrode Hielscher UIS250V (Hielscher Ultrasonics GmbH, Teltov, Germany) and magnetic stirring. The surfactant content was chosen as per manufacturer guidelines (0.5 vol.%), while Table 1 shows the actual amount used after rounding the numbers. The prepared solution and dispersion were combined and homogenized using magnetic stirring for 2 h at 62 °C. The resulting solution (about 150 mL) was cast in a Petri dish in a fume hood and left to evaporate overnight. Any leftover solvent was removed by vacuum drying (24 h at 70 °C). Composite films for testing were prepared using compression molding (135 °C, 5 min) (Carver Inc., Wabash, IN, USA), followed by rapid cooling between steel plates to room temperature (22 °C). For light flash analysis and broadband dielectric spectroscopy, 1.0 mm thick samples were used; 0.5 mm thick samples were used for MHz and THz spectroscopy, and 0.1 mm thick samples were used for four-point surface resistivity. The sample abbreviations and the volume and weight concentrations of the fillers and surfactant are given in Table 1.

### 2.3. Characterization

The densities of the PBS composites were determined using the hydrostatic displacement method by measuring sample weight in air and ethanol ($d_{\mathrm{EtOH}}$ = 0.805 g/cm^3^) on Sartorius KBBA 100 (Sartorius AG, Göttingen, Germany) analytical scales equipped with a hydrostatic density measurement kit Sartorius YDK 01. Density [d (g/cm^3^)] was calculated according to the following equation:

(1) $$d=\frac{m_{a}\left(d_{\mathrm{EtOH}}-0.0012\right)}{0.99983\left(m_{a}-m_{s}\right)}+0.0012$$

where $m_{a}$ (g) is the sample’s mass in air, $m_{s}$ is the sample’s apparent mass as measured when submerged in ethanol (g), and $d_{\mathrm{EtOH}}$ is the density of ethanol (0.805 g/cm^3^), which was determined with a hydrometer.

Thermal diffusivity and thermal conductivity measurements were obtained using a light flash apparatus (LFA) Netzsch 447 NanoFlash (NETZSCH-Gerätebau GmbH, Selb, Germany) according to EN ISO 22007-4. The square-shaped samples with an edge length of 12.7 mm and a thickness of 1 mm were spray coated with a graphite-based coating Graphit 33 (Kontakt Chemie, Zele, Belgium) in order to ensure equal opacity and absorbance. The spray coating was applied only for thermal diffusivity and thermal conductivity measurements. The samples’ heat capacities were obtained compared to a Pyrex sample with a known heat capacity. In addition, the samples were subjected to five parallel thermal diffusivity measurements at 25, 35, and 45 °C.

Thermal conductivity [λ (W/mK)] was calculated according to the following equation:

(2) λ(T)=a(T)d(T)Cp(T)

where a is thermal diffusivity (mm^2^/s), d is the sample density (g/cm^3^), Cp is the sample-specific heat capacity (J/(gK)), and T is the absolute temperature (K).

The thermal conductivity activation energies [$E_{a}\;$ (eV)] of the samples were calculated according to the Arrhenius equation [21]:

(3) $$\lambda =\lambda_{0}e^{\left(-\frac{E_{a}}{kT}\right)}$$

where λ is the thermal conductivity (W/mK), $\lambda_{0}$ is the extrapolated inherent thermal conductivity at infinite temperature (W/(mK)), T is the absolute temperature (K), and k is the Boltzmann constant (8.617 × 10^−5^ eV/K).

In the low-frequency range from 10^−2^ to 4 × 10^7^ Hz, AC conductivity [σ’ (S/m)] and real (ε′) and imaginary parts of permittivity (ε″) were obtained using a (DS) Novocontrol BDS 50 (Novocontrol Technologies GmbH & Co., KG, Montabaur, Germany) broadband dielectric spectrometer. Disc-shaped samples with a thickness of 1 mm and a diameter of 30 mm were placed between plate electrodes and measured at room temperature.

An Agilent 8714ET vector network analyzer (Agilent Technologies Inc., Santa Clara, CA, USA) was used for measurements in the frequency range 1 MHz–3 GHz.

In the microwave frequency range 8–12 GHz and 26–40 GHz, waveguide spectrometers were used. Disc-shaped samples with a thickness of 0.5 mm were placed in a waveguide holder. The dielectric properties were calculated according to the method described in Reference [22]. The measurements were determined to be accurate to within 10%. In the 3–8 GHz and 12–18 GHz ranges, linear approximation was used.

In the terahertz frequency range from 0.01 to 3 THz, a terahertz time-domain spectrometer (EKSPLA UAB, Vilnius, Lithuania) based on a femtosecond laser (1 µm wavelength, pulse duration under 150 fs) was used for the spectrum measurements. In addition, a photoconductive terahertz emitter–detector based on GaBiAs was used. The noise-to-signal ratio was the highest (60 dB) at 0.5 THz, and the accuracy was within 1%. The thickness of all of the samples for THz investigations was 0.5 ± 0.1 mm.

The sheet resistance of the PBS composites was determined using a four-point probe Signatone S-302-4 (Signatone Corporation, Gilroy, CA, USA) and an electrical source measure unit Keithley 2450 (Keithley Instruments, Llc., Cleveland, OH, USA). The square-shaped films with a thickness of 100 μm and an edge length of 60 mm were tested in a current range of 10 nA to 10 mA with a maximum voltage of 21 V until the measured resistance value stabilized. Measurements were repeated a minimum of 5 times for each sample. As a result, the sheet resistance was calculated as follows:

(4) $$R_{s}=4.5324\frac{U}{I}f_{1}f_{2}\;$$

where $R_{s}$ is the electrical sheet resistance (Ω/sq), 4.5324 is the correction factor determined by the model of the four-point probe, according to the technical data by Signatone, U is the measured voltage (V), I is the applied current (A), $f_{1}$ is the correction factor for the sample thickness, and $f_{2}$ is the correction factor for the sample geometry ($f_{2}$ = 1 as the probe spacing is magnitudes of order smaller than the sample size).

The correction factor for sample thickness was calculated as follows:

(5) $$f_{1}=\frac{\ln\left(2\right)}{\ln\left[\frac{\sinh\left(\frac{t}{s}\right)}{\sin h\left(\frac{t}{2s}\right)}\right]}$$

where t is the thickness of the sample (100 μm), and s is the probe spacing (1000 μm).

A response surface for a given target function in coded factors is generally written as follows:

(6) $$Y\;=b_{0}{+b}_{1}X_{1}{+b}_{2}X_{2}{+b}_{12}X_{1}X_{2}{+b}_{11}X_{1}^{2}{+b}_{22}X_{2}^{2}$$

where b_i_ are the regression coefficients, which are calculated according to Equations (7)–(10), and $X_{1}$ (for MWCNT filler) and $X_{2}$ (for Fe_3_O_4_ filler) are coded factors which are calculated according to Equation (11):

(7) $$b_{1}=\frac{\sum_{i=0}^{n}Y_{i}\cdot X_{1}}{\sum_{i=0}^{n}X_{1}^{2}}$$

(8) $$b_{2}=\frac{\sum_{i=0}^{n}Y_{i}\cdot X_{2}}{\sum_{i=0}^{n}X_{2}^{2}}$$

(9) $$b_{12}=\frac{\sum_{i=0}^{n}Y_{i}\cdot X_{1}\cdot X_{2}}{\sum_{i=0}^{n}X_{1}^{2}\cdot X_{2}^{2}}$$

(10) $$\{\begin{array}{c} n\cdot b_{0}+\sum_{i=0}^{n}X_{1}^{2}\cdot b_{1}+\sum_{i=0}^{n}X_{2}^{2}\cdot b_{22}=\sum_{i=0}^{n}Y_{i}\\ \sum_{i=0}^{n}X_{1}^{2}\cdot b_{0}+\sum_{i=0}^{n}X_{1}^{4}\cdot b_{11}+\sum_{i=0}^{n}X_{1}^{2}\cdot X_{2}^{2}\cdot b_{22}=\sum_{i=0}^{n}Y_{i}\cdot X_{1}^{2}\\ \sum_{i=0}^{n}X_{2}^{2}\cdot b_{0}+\sum_{i=0}^{n}X_{1}^{2}\cdot X_{2}^{2}\cdot b_{11}+\overset{n}{\underset{i=0}{\sum }}X_{2}^{4}\cdot b_{22}=\sum_{i=0}^{n}Y_{i}\cdot X_{2}^{2} \end{array}$$

(11) $$X_{1},\;X_{2}=\frac{x-\left(\frac{x_{max}+x_{min}}{2}\right)}{\left(\frac{x_{max}-x_{min}}{2}\right)}$$

where x is the filler concentration in each composite, $x_{max}$ is the maximum concentration of a filler in the series (0.6 or 12 accordingly), and $x_{min}$ is the minimum concentration of a filler in the series (0.1 or 8 accordingly).

The structure of a nanocomposite fracture surface (a fracture in liquid nitrogen) was examined using an FEI Nova NanoSEM 650 Schottky field emission scanning electron microscope (FESEM, Hillsboro, OR, USA). A voltage of 10 kV was used to examine the morphology. Surface coatings were not applied.

The electromagnetic shielding properties of the nanocomposites in the microwave range were calculated according to the following equations [23,24]:

(12) $$\{\begin{array}{c} S_{11}=\frac{-j\left[{\left(\frac{k_{z}}{k_{2z}}\right)}^{2}-1\right]\sin\left(k_{2z}\tau \right)}{2j\left(\frac{k_{z}}{k_{2z}}\right)\cos\left(k_{2z}\tau \right)+\left[{\left(\frac{k_{z}}{k_{2z}}\right)}^{2}+1\right]\sin\left(k_{2z}\tau \right)}\\ S_{21}=\frac{2\left(\frac{k_{2z}}{k_{z}}\right)}{-2\left(\frac{k_{2z}}{k_{z}}\right)\cos\left(k_{2z}\tau \right)+j\left[{\frac{k_{2z}}{k_{z}}}^{2}+1\right]\sin\left(k_{2z}\tau \right)} \end{array}$$

where $S_{11}$ is the reflection coefficient; $S_{21}$ is the transmission coefficient; $k_{z}=\frac{2\pi }{\lambda }$ and $k_{2z}=\frac{2\pi \varepsilon^{2}}{\lambda }$ are wave numbers in the vacuum and the sample’s media, correspondingly; and τ is the thickness of the layer. The absorption (A) of the layer was calculated according to the following equation:

(13) $$A=1-{\left(S_{11}\right)}^{2}-{\left(S_{21}\right)}^{2}$$

## 3. Results and Discussion

### 3.1. Structure

The prepared nanocomposite filler–filler and matrix–filler compatibility and preparation method suitability were analyzed with the density measurements. Table 2 shows the experimental density (d_EX_) and density calculated according to the rule of mixtures (d_TH_) of nanocomposites and includes the calculated void percentage (V_p_) in the nanocomposites structure (percentage difference between the measured and calculated density values). The inclusion of iron nanoparticles significantly increases the density values of all of the prepared nanocomposites. Besides the two compositions, nanocomposites show a relatively low void percentage with values below 1%. Thus, the filler–filler and matrix-filler compatibility are relatively good, as density measurements do not indicate major structural defects.

**Figure 1:**
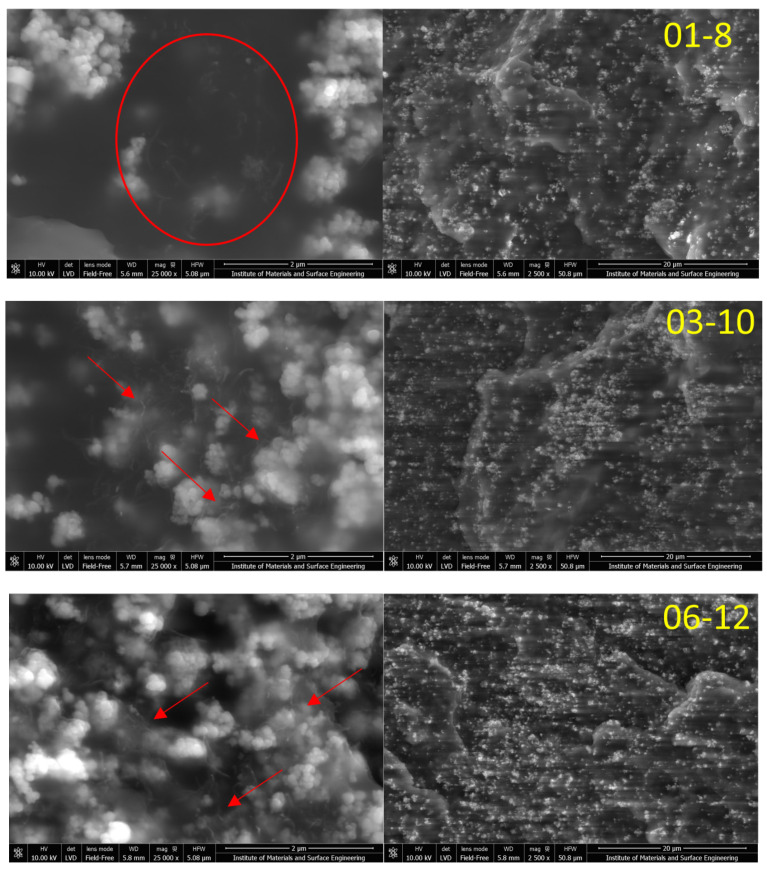
SEM micrographs for the selected nanocomposites using 2500× and 25,000× magnification. Arrows indicate cases of clearly distinguishable separate MWCNTs.

Further structure analysis was performed with scanning electron microscopy (SEM), which allows for the investigation of filler interactions, agglomeration, and dispersion to a higher extent. SEM micrographs of the three selected nanocomposites (01-8, 03-10, and 06-12) are presented in Figure 1. A magnification of 2500× was selected in order to observe nanoparticle distribution. The filler particles were well distributed in the PBS matrix, covering the fracture surface. It can be seen that the iron nanoparticles formed particle clusters (agglomerates) with a size below 1 μm and can be easily spotted at relatively low magnification. The formation of iron oxide nanoparticle clusters is typical with larger nanoparticles (above 20 nm) and occurs as a result of the attractive magnetic dipole–dipole interactions [25] and electrostatic interactions [26]. At the same time, it is also promoted by the relatively high filler loadings used (i.e., 27 wt.% to 36 wt.%). Clusters are also classified in the literature as a form of iron oxide nanoparticle morphology [27,28,29,30]; thus, the achieved morphology can be regarded as reasonably well dispersed for iron oxide nanoparticles. With a higher magnification of 25,000×, MWCNTs can be observed. For all three selected nanocomposites, well-distributed and non-agglomerated MWCNT are visible.

Nevertheless, two distinct cases of MWCNT arrangement can be seen in the micrographs. For 01-8, MWCNT form their own distinct network in the PBS matrix (well dispersed), while, for composites 03-10 and 06-12, MWCNT can be seen on the surface of iron oxide nanoparticles, encompassing the clusters. At these loadings, synergy in the form of the structural arrangement of MWCNT on the iron oxide surface is observed. The synergy in morphology should contribute to changes in the electromagnetic and thermal properties of the material.

### 3.2. Light Flash Analysis

**Figure 2:**
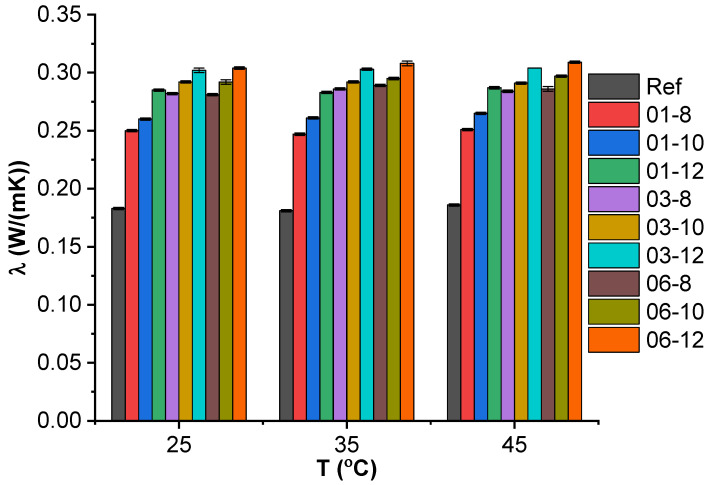
Thermal conductivity of hybrid composites at 25, 35, and 45 °C.

Thermal conductivity values from light flash analysis at 25, 35, and 45 °C can be seen in Figure 2. The thermal conductivity dependence on filler content at 25 °C is presented as a surface plot in Figure S1. The reference system (PBS) has a thermal conductivity of 0.181 W/(mK) at 25–45 °C, which is about 0.06 W/(mK) lower than the values reported for pure PBS by Yin et al. [31]. However, there are likely to be differences between separate grades and sources of PBS, and our reference system contains a surfactant. Therefore, our measured values are in line with the expected results. The highest thermal conductivity values can be observed for 06-12 (0.304–0.309 W/(mK), 68% improvement over neat system) and 06-10 (0.292–0.297 W/(mK), 66% improvement) systems, which correspond to the highest loadings of nanofillers used. Two distinct regions are visible in the surface plot (Figure S1). First, at 0.1 vol.% MWCNT, thermal conductivity rapidly increases with the increase of Fe_3_O_4_ content. Second, at 0.3–0.6 vol.% MWCNT, where the impact of MWCNT loading diminishes (a percolation network is already formed) and the thermal conductivity is complemented by the Fe_3_O_4_ content.

Thermal carriers flow along the routes of least resistance [32], which seem to be provided both by the percolated MWCNT network and the crystalline Fe_3_O_4_ nanoparticles that connect highly ordered regions. When the interfacial contact resistance between the MWCNT network and Fe_3_O_4_ phases is small, phonons are more likely to propagate through the highly ordered Fe_3_O_4_ phase. At 0.3 vol.% MWCNT, the contact between the separate phases seems to reach a maximum, determined by the surface area of Fe_3_O_4_ that is available to MWCNT. Given the increase in Fe_3_O_4_ content, thermal conductivity increased linearly at 0.3 vol.% and 0.6 vol.% MWCNT, indicating adequate filler dispersion and low defect concentration. This aligns with the void percentage calculations and SEM micrographs discussed previously, as well as with the correlation between the MWCNT dispersion and thermal properties observed in a previous study [33].

The thermal conductivity value can also be significantly influenced by the crystallinity of the polymer matrix, which is likely impacted by the addition of a surfactant and the inclusion of fillers, which has been widely attested to in the literature [34,35,36,37,38,39,40,41,42]. The heterogeneous nucleation effects are not discussed in this paper.

### 3.3. Activation Energy of Thermal Conductivity

**Figure 3:**
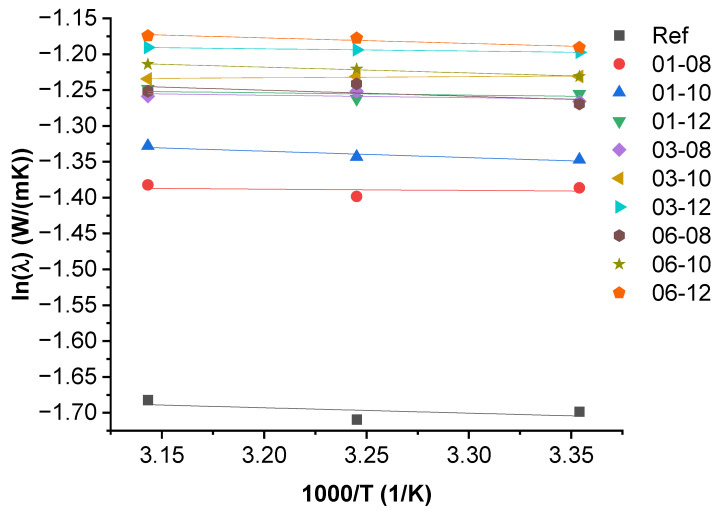
Arrhenius plots of PBS hybrid composites.

Thermal conductivity Arrhenius plots are shown in Figure 3. The numerical values of the activation energy ($E_{a}$), the minimum energy necessary for a polymer composite system to overcome a thermally activated potential barrier, and the inherent thermal conductivity ($\lambda_{0}$) at infinite temperature are summarized in Table S3. Thermal conductivity values have a very low or no dependency on the temperature within the chosen temperature amplitude. The combination of several effects could explain this: first, the decrease of the phonon mean free path (diffusion) caused by the increase of the specific heat capacity of the crystalline phases in PBS, MWCNT, and Fe_3_O_4_ due to the Umklapp scattering [43]; second, the effect of the increased probability of phonon transfer between PBS molecules with temperature increase due to thermal oscillation in the amorphous phase [44]; third, thermally activated electron transport is likely to contribute relatively little to the overall thermal conductivity due to the low concentration of MWCNT filler [45]; and finally, the inherent thermal conductivity depends upon the available thermally activated carriers, the dispersion of the nanofiller particles, and the interaction between the components (phonon scattering on the interfaces of fillers and the matrix) [32,44,46,47].

### 3.4. Surface Resistivity Measurements and Response Surface Analysis

**Figure 4:**
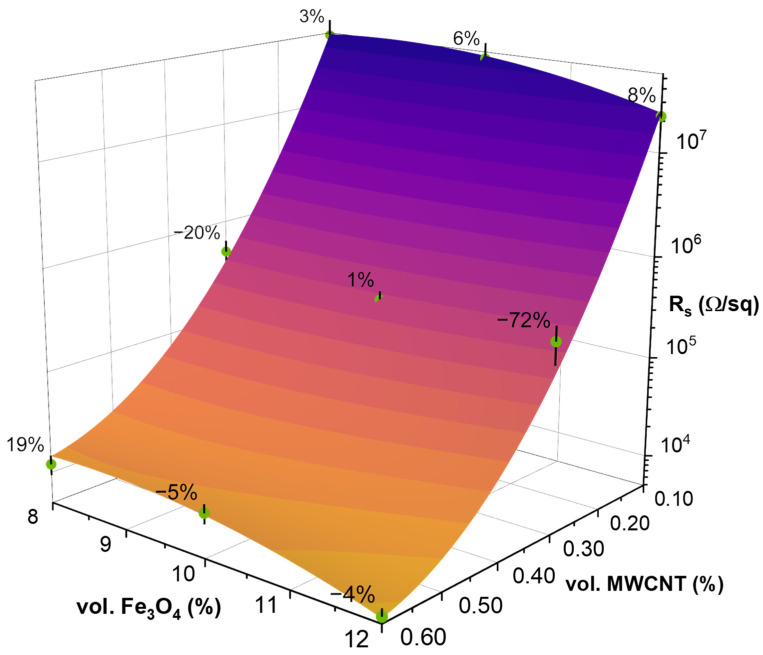
Experimental average surface resistivity values (in green) with error bars (in black), and modelled surface plot for PBS hybrid nanocomposites, with percentage values signifying the deviation of the model from the experimental data.

Figure 4 displays the surface resistivity values obtained via four-point probe resistivity measurements and the calculated approximate response surface model according to Equation (6). Table S1 contains the coded factors used for the modelling, calculated according to Equation (11) and the experimentally determined $R_{s}$ values used as entry conditions. Table S2 contains the regression coefficients calculated by Equations (7)–(10) and their adjusted values. The highest accuracy in the adjusted surface response model can be seen in the 01-8/03-10/06-12 axis, while the highest deviation can be observed near the 03-12 system. The adjustments were introduced to bring the surface within the bounds of the absolute error values of the experimental data.

As expected, the lowest surface resistivity values can be seen in the 06-12 system, reaching 7.6 × 10^3^ Ω/sq, due to the formation of a percolated MWCNT structure. Comparing the fillers, the most substantial influence on the surface resistivity is from MWCNT, where the increase of filler content from 0.1 to 0.6 vol.% contributes to a decrease in surface resistivity by 3.6 orders of magnitude (from values above 10^7^ Ω/sq to values below 10^4^ Ω/sq) between 01-12 and 06-12, which signifies the ongoing percolation and densification of the MWCNT network. The precise percolation threshold of CNT in a given matrix depends on the level of composite processing [48], CNT morphology, and matrix used. Our value is in line with the percolation threshold of MWCNT (0.25–0.50 wt.%) determined by Pedroni et al. for styrene-butadiene-styrene block copolymers [49]. The Fe_3_O_4_ content minimizes the surface resistivity values, which are mostly within the measurement error. Between 10 and 12 vol.% Fe_3_O_4_ loadings, a decrease of 0.37 orders of magnitude can be observed for 06-10 and 06-12. Meanwhile, between 06-8 and 06-10, there is an increase in surface resistivity of 0.04 orders of magnitude. This trend could signify a critical value of Fe_3_O_4_ around 10 vol.%. This could be attributed to the distribution of Fe_3_O_4_ on the surface of the composite, forming a 2D percolated structure that contributes to the conductivity of the composite. The surface resistivity of the reference system is omitted, as it is above 10^13^ Ω/sq and could not be accurately measured with the current measurement system.

### 3.5. Broadband Dielectric Spectroscopy

**Figure 5:**
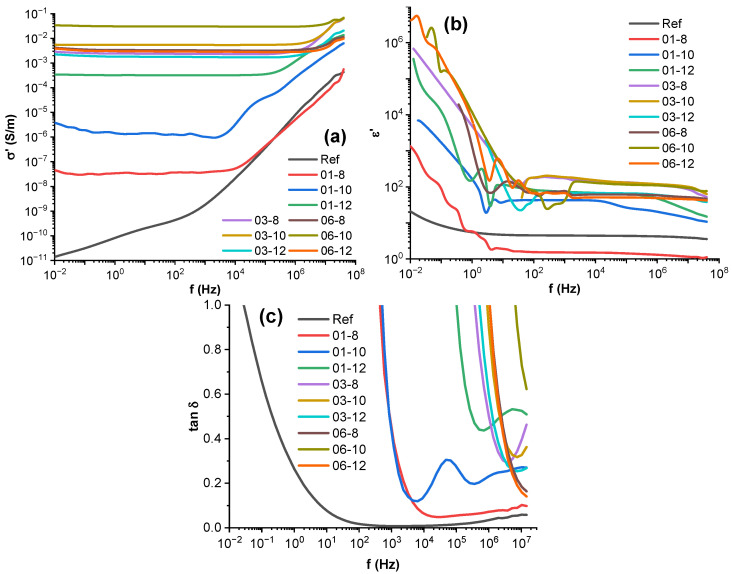
The broadband dielectric spectroscopy (**a**) AC conductivity, (**b**) real part of permittivity, and (**c**) loss tangent of permittivity of the PBS hybrid nanocomposites.

Figure 5a shows the AC broadband electrical conductivity for PBS hybrid nanocomposites. The reference system displays a conductivity of 8.12 × 10^−11^ S/m at 1 Hz, which is slightly higher but comparable to the values (1.72 × 10^−11^ S/m) reported by Ohki et al. [4]. The hybrid nanocomposites show frequency-independent conductivity up to a specific frequency where the slope rapidly changes, which is in line with Jonscher’s law and the universal dielectric response [50]. The reference composition was the only one not to achieve frequency-independent conductivity due to a lack of charge carriers. Depending on the filler content, frequency dependence begins at different frequencies (this frequency can be called the critical frequency). For the 01-8 system, frequency dependence starts at around 10 kHz. The 01-10 system shifts to frequency-dependent behavior at around 3.2 kHz, showing a secondary slope change at around 47 kHz. The 01-12 system shows frequency dependence starting at 250 kHz, with a slight slope change at 5 MHz. As observed previously, the critical frequency increases together with the conductivity at low frequencies according to the percolation theory [51,52].

A comparison can be made for the impact of the fillers on the AC conductivity of composites with 0.1 vol.% MWCNT loading. An increase in Fe_3_O_4_ content displays improvements in electrical conductivity over several magnitudes (from 3 × 10^−8^ S/m for 8 vol.% to 3.2 × 10^−4^ S/m for 12 vol.%). However, in 0.3 and 0.6 vol.% MWCNT hybrid systems, increases in Fe_3_O_4_ content yield only small conductivity changes, with the 06-10 system being an outlier, achieving the highest conductivity out of all composites (0.03 S/m). Further increases in MWCNT content above 0.3 vol.% have a diminished effect on higher conductivity, remaining within 10^−3^–10^−2^ S/m. This can be explained by the development of a percolated MWCNT network between 0.1 and 0.3 vol.%, which is in line with the previous discussion on $R_{s}\;$, as well as increases in Fe_3_O_4_, aiding the development of a percolated network in the 0.1 vol.% MWCNT systems. This is possible either due to Fe_3_O_4_ particles forming their network at 10 and 12 vol.% or Fe_3_O_4_ particles and clusters displacing free polymer volume. Thus, aiding in the densification of the MWCNT-filled polymer phase, it causes percolation according to Essam’s percolation theory [53]. Nanocomposite electrical percolation has been elaborated on in our previous paper on PBSA-carbon nanocomposites [54]. In composites near the percolation threshold, electron transport and conduction are caused mainly by electron tunnelling over small distances. Above the percolation threshold, a conductive network has formed and ohmic contact conductivity dominates [55].

The frequency-dependent real part of permittivity and dielectric dissipation tan(δ) of the composites in the spectrum frequency range from 10^−2^ to 10^8^ Hz are presented in Figure 5b,c, respectively. Two separate regions for the real part of permittivity can be observed for filled composites [56]. At frequencies from 10^−2^ to 10 Hz, a drastic drop in the real part of permittivity over four orders of magnitude can be seen. The interfacial polarization of interfaces between the non-conductive polymer and conductive fillers mainly causes the high values. In the second region, the real part of permittivity becomes somewhat monotonous, with a tendency to decrease at higher frequencies. The reference system shows a response characteristic to dielectric polymer materials, with the real part of permittivity of 4.4 at 1 kHz.

The tan(δ) values of the composites are highly dependent on their electrical conductivity, which effectively dissipates electrical energy and other relaxation processes. Large drops in tan(δ) can be seen close to the transition of frequency-dependent electrical conductivity, shifting towards the universal dielectric response. For the 01-10 system, an additional relaxation process can be seen at around 10 kHz, which signifies a shift in the conduction mechanism in Fe_3_O_4_ particles. At lower frequencies the tunnelling of polarons (long-range charge mobility), with subsequent grain boundary polarization, dominates [57]. However, at around 10 kHz, a shift to electron hopping (short-range mobility) occurs and is combined with electrons moving within the crystal structure of Fe_3_O_4_ between Fe^3+^ and Fe^2+^ ions. This effect and other electrical and dielectric properties of Fe_3_O_4_ nanoparticles have been investigated in detail by Radoń et al. [57]. For the 01-12 system, a tan(δ) peak can be observed at around 5 MHz. However, it is unclear whether a similar process causes it. For the 0.3 and 0.6 vol.% MWCNT-filled systems, a shift can be seen at around 10 MHz, which is attributed to artefacts near the limit of the frequency resolution of the testing apparatus.

### 3.6. High-Frequency Spectroscopy, Terahertz Transmittance, EMI Shielding Efficiency

**Figure 6:**
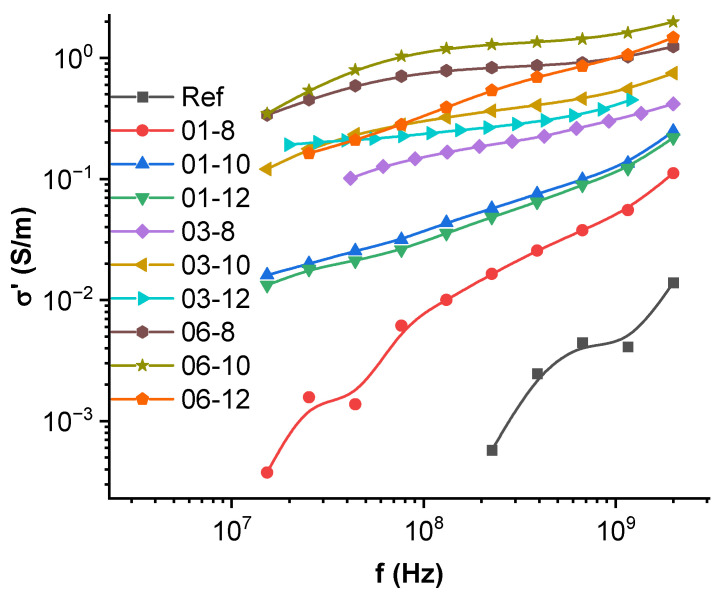
AC conductivity in the high-frequency VHF-L bands for PBS hybrid nanocomposites.

**Figure 7:**
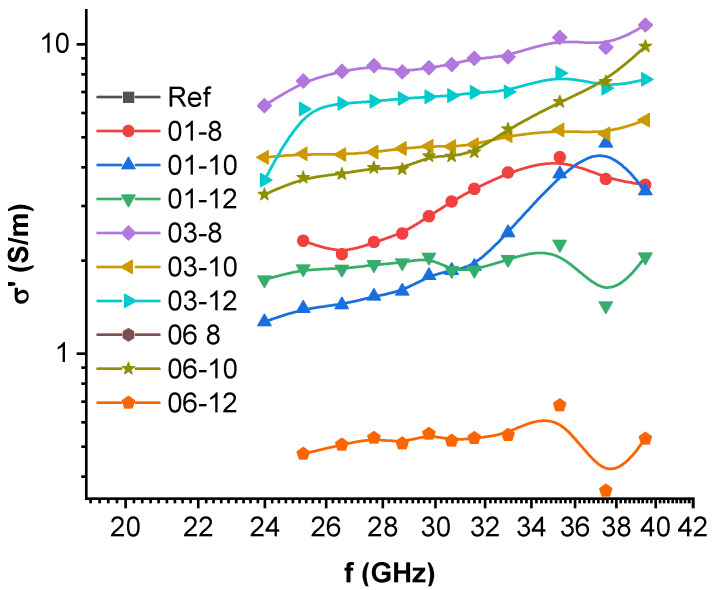
AC conductivity in the microwave K-Ka bands for PBS hybrid nanocomposites.

AC conductivity measurement results for high frequencies and microwave K-Ka bands are presented in Figure 6 and Figure 7. Regarding the VHF-L band conductivity measurements (Figure 6), a uniform response of increasing conductivity with increasing frequency can be observed. Following low-frequency conductivity measurements (Figure 5a), composites with the highest MWCNT fraction exhibit the highest conductivity values. The highest conductivity (1.98 S/m) can be seen in the 06-10 system at 2 GHz. It can also be seen that 10 vol.% Fe_3_O_4_ filled systems have slightly higher conductivity values than 8 and 12 vol.% systems with the same MWCNT content. This could be explained by a critical volume of Fe_3_O_4_, which contributes as a conductive filler while not diminishing the existing percolated network of MWCNT. In the 8–10 vol.% range, Fe_3_O_4_ contributes to the conductivity of a composite, while in the 10–12 vol.% range, it starts to become detrimental. The best example of this effect is in the comparison of 0.1 vol.% MWCNT systems, where there is a rapid increase in the conductivity between 01-8 and 01-10 (denser percolated network) and a slight diminishment between 01-10 and 01-12 (a larger number of interfaces that impede conduction).

In the 25–40 GHz range (Figure 7), composite conductivity slightly increases with increasing frequency. The highest conductivity can be seen in the 03-8 system (11.54 S/m) at 40 GHz. A clear correlation between the filler concentration and conductivity cannot be seen, except for the reference system, which had the lowest conductivity. The conductivity values fluctuate over the measurement range, which points to other underlying processes that might be more related to the overall structure and morphology of the conductor networks within the composite. The incident electromagnetic (EM) radiation is likely to interact with the electrically conductive MWCNT and ferrimagnetic Fe_3_O_4_ in a complex way that impacts the measurement result. The low conductivity of the 06-12 sample could be related to structural defects.

**Figure 8:**
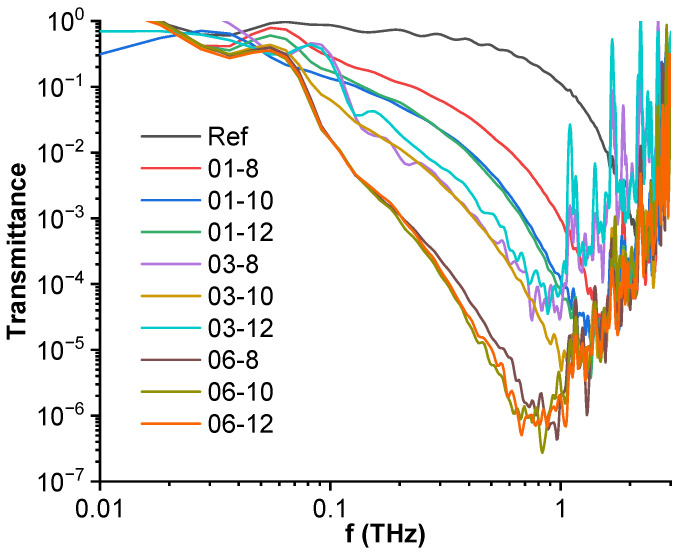
Time domain spectroscopy transmittance spectra for PBS hybrid nanocomposites.

The time domain spectroscopy transmittance spectra for PBS hybrid nanocomposites can be seen in Figure 8. The tested nanocomposites show a drop in transmittance by up to six orders of magnitude in the terahertz range. A clear inverse correlation between the MWCNT filler fraction and transmittance can be seen, with the 0.6 vol.% MWCNT filled samples displaying the least transmittance with a peak at around 0.7 to 1 THz. The transmittance peak is seen to move in the direction of lower frequencies with the increase of filler content. A frequency-dependent region starting at 1 THz is an artifact due to the absence of incident and transmitted signals. Fe_3_O_4_ filler content displays the highest influence in the 0.1 vol.% MWCNT systems, where the increase from 8 to 10 vol.% Fe_3_O_4_ causes a denser MWCNT percolated structure to form, resulting in lower transmittance. The impact of Fe_3_O_4_ is lessened in the 0.3 and 0.6 vol.% systems as they are already above the percolation threshold.

**Figure 9:**
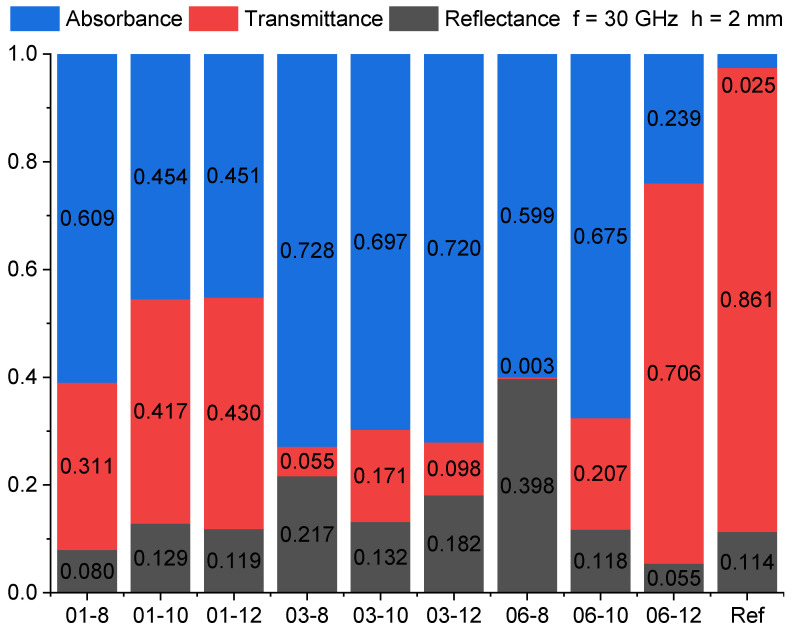
PBS hybrid nanocomposite EM absorbance, transmittance, and reflectance at 30 GHz for 2 mm-thick samples.

Figure 9 represents the EM absorbance, reflectance, and transmittance for PBS hybrid nanocomposites at a frequency of 30 GHz. Compared to the reference system, hybrid nanocomposites exhibit high levels of absorbance, which are higher than the transmitted values. The highest absorbance was seen in the 03-8 system (72.8%). EM radiation is likely to be absorbed and dissipated as heat at the interfaces of electric and dielectric phases, as well as reflected from electric components (such as MWCNT) and dissipated further by multiple reflections [58].

The highest shielding effectiveness (signal attenuation expressed in dB) can be seen in the 06-8 (52.0 dB), 03-8 (25.6 dB), and 03-12 (20.5 dB) systems. The 06-8 system can be considered particularly good compared to other composites in the literature with much higher loading factors. Sushmita et al. developed a multi-layer foamed composite that combined poly(vinylidene fluoride) (PVDF), polyurethane, CNT, and polycarbonate, achieving a shielding effectiveness of 30 to 40 dB in the Ku and K bands [59]. Tsonos et al. presented a highly loaded PVDF, 15 wt.% Fe_3_O_4_, and 7 wt.% CNT composite that displayed a 25–30 dB loss in the C and X bands [60]. Kuzhir et al. described epoxy-MWCNT composites and carbon foams that reached 2–23.4 dB shielding effectiveness at 30 GHz [61].

**Figure 10:**
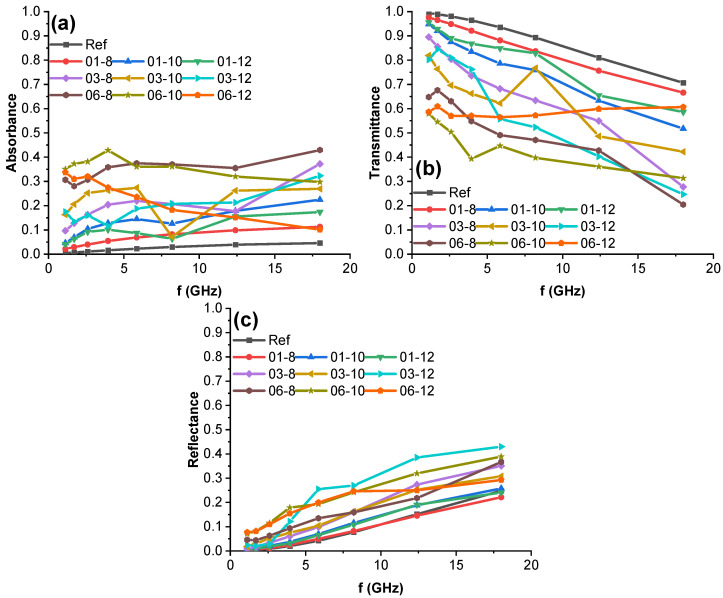
PBS hybrid nanocomposite EM (**a**) absorbance, (**b**) transmittance, and (**c**) reflectance at frequencies from 1.12 to 18 GHz for 1 mm thick samples.

The EM absorbance, reflectance, and transmittance spectra of PBS hybrid nanocomposites at frequencies from 1.12 to 18 GHz are presented in Figure 10. The transmittance (Figure 10b) and reflectance (Figure 10c) spectra of the composites seem to follow a somewhat linear trend, with the transmittance decreasing at higher frequencies due to increased reflectance. Absorbance (Figure 10a) is relatively constant with a slight trend to increase at higher frequencies. The 06-12 system is an outlier in the absorbance and transmittance graphs, with the transmittance staying constant (0.55–0.60) over the entire range due to absorbance decreasing at the same rate as reflectance increases. This effect could be caused by defects in the MWCNT percolated network at higher Fe_3_O_4_ loadings, as discussed previously. The highest absorbance can be seen in the 06-8 and 06-10 nanocomposites, with 06-8 being a better absorber at higher frequencies and 06-10 at lower frequencies. The highest reflectance was achieved in the 03-12 system (0.40–0.45) at 18 GHz. The 01-8 system displays spectra that are very similar to the reference system, indicating that higher filler contents are necessary. Overall, MWCNT content correlates with increased absorbance, especially at or above 0.3 vol.%, whereas Fe_3_O_4_ content provides synergistic EMI shielding effects up to 12 vol.%, whereupon reflectance starts to increase.

## 4. Conclusions

Poly(butylene succinate) hybrid multi-walled carbon nanotube/iron oxide nanocomposites were successfully prepared with a very good filler dispersion, as indicated by SEM analysis. Iron oxide particles formed particle clusters, while MWCNTs formed percolated networks. With the addition of at least 0.3 vol.% MWCNT, it was possible to observe MWCNT distribution on the surface of iron oxide particle clusters and the formation of an interconnected hybrid filler network.

Electrical and thermal conductivity were mainly affected by the content of MWCNT filler. After reaching the MWCNT percolation threshold at around 0.3 vol.%, further increases in iron oxide nanoparticle concentration had a reduced effect on the electrical and thermal conductivity of composites. The highest frequency-independent AC electrical conductivity (from 10^−2^ to 10^7^ Hz) was reached upon adding 0.6 vol.% MWCNT and 10 vol.% Fe_3_O_4_ (with a value of around 3.1 × 10^−2^ S/m). Thermal properties, electrical conductivity, and surface resistivity favored compositions with the highest MWCNT loading (0.6 vol.%). A clear property saturation effect could be seen between 10 and 12 vol.% Fe_3_O_4_ composites, impacting the structure of the MWCNT percolated network at the higher Fe_3_O_4_ loadings. Transmittance was reduced to around 0.3% (SE of 52 dB) in the microwave frequency of 30 GHz, while in the 0.1 to 1.0 THz range, it was reduced by up to 6 orders of magnitude compared to the reference system. In the 1.12 to 18 GHz range, a highly variable absorbance-reflectance response could be observed while maintaining relatively low transmittance values (20–30%). The 06-8 composite shows the lowest overall transmittance values at higher frequencies, while the 06-10 composite has a higher performance at lower frequencies.

The presented nanocomposites are suitable for use in EMI shielding applications and offer a novel way to increase absorbance and reduce transmittance by exploiting synergistic hybrid composite morphology. The additional electrical conductivity, surface resistivity, and thermal conductivity improvements and their accompanying discussion show potential for nanocomposite use within the fields of ESD, anti-static, and thermally conductive materials. The biodegradable PBS matrix allows for the creation of novel, more ecologically friendly, high-performance EMI shielding composite materials.

For future research, the authors see several options to expand the present topic, which include but are not limited to thermal conductivity modeling, studies in phase formation, and more complex matrix–filler interactions. Filler distribution (and synergistic morphology) is highly influenced by the processing method chosen; therefore, it would be fruitful to explore the impacts of melt-mixing conditions on the EMI shielding and thermal performance.

## Figures and Tables

**Table 1:** Filler and surfactant concentrations.

No.	Sample	MWCNT (vol.%)	MWCNT (wt.%)	Fe_3_O_4_ (vol.%)	Fe_3_O_4_ (wt.%)	Surfactant (vol.%)	Surfactant (wt.%)
1	Ref	0.00	0.00	0.00	0.00	0.51	0.45
2	01-8	0.10	0.12	8.20	26.55	0.50	0.35
3	01-10	0.10	0.11	10.24	31.59	0.51	0.34
4	01-12	0.10	0.11	12.28	36.17	0.54	0.34
5	03-8	0.30	0.36	8.20	26.52	0.51	0.36
6	03-10	0.30	0.34	10.24	31.57	0.50	0.34
7	03-12	0.30	0.32	12.28	36.16	0.50	0.32
8	06-8	0.60	0.72	8.20	26.49	0.50	0.35
9	06-10	0.60	0.69	10.24	31.52	0.53	0.35
10	06-12	0.60	0.65	12.28	36.11	0.50	0.32

**Table 2:** Nanocomposite density and void percentage.

Sample	d_EX_ (g/cm^3^)	d_TH_ (g/cm^3^) *	V_p_ (%)
Ref	1.264 ± 0.0011	1.259	–
01-8	1.566 ± 0.0059	1.575	0.57
01-10	1.618 ± 0.0082	1.653	2.12
01-12	1.717 ± 0.0064	1.731	0.81
03-8	1.562 ± 0.0059	1.576	0.89
03-10	1.649 ± 0.0057	1.654	0.30
03-12	1.708 ± 0.0068	1.733	1.44
06-8	1.564 ± 0.0068	1.578	0.89
06-10	1.652 ± 0.0034	1.656	0.24
06-12	1.724 ± 0.0062	1.734	0.58

* The densities of the constituents (PBS, MWCNT and iron oxide) are given in Section 2.2.

## Data Availability

The data presented in this study are available on request from the corresponding authors.

## Supplementary Materials

The following supporting information can be downloaded at: https://www.mdpi.com/article/10.3390/polym15030515/s1, Figure S1: Surface plot of thermal conductivity of hybrid composites at 25 °C, Table S1: Coded factors and initial Y values for the surface response model of Rs, Table S2: Calculated and adjusted regression coefficients for the response surface model of Rs, Table S3: Parameters of the Arrhenius equation for PBS hybrid nanocomposites.
