# Supplementary material for: Poly(Butylene Succinate) Hybrid Multi-Walled Carbon Nanotube/Iron Oxide Nanocomposites: Electromagnetic Shielding and Thermal Properties
Source: Polymers (Basel). 2023 Jan 18;15(3):515. doi: 10.3390/polym15030515 (PMC9921677; doi:10.3390/polym15030515)
Supplement: Supplementary file 1 [file polymers-15-00515-s001.zip › polymers-2144067-supplementary.pdf]

Supplementary Materials

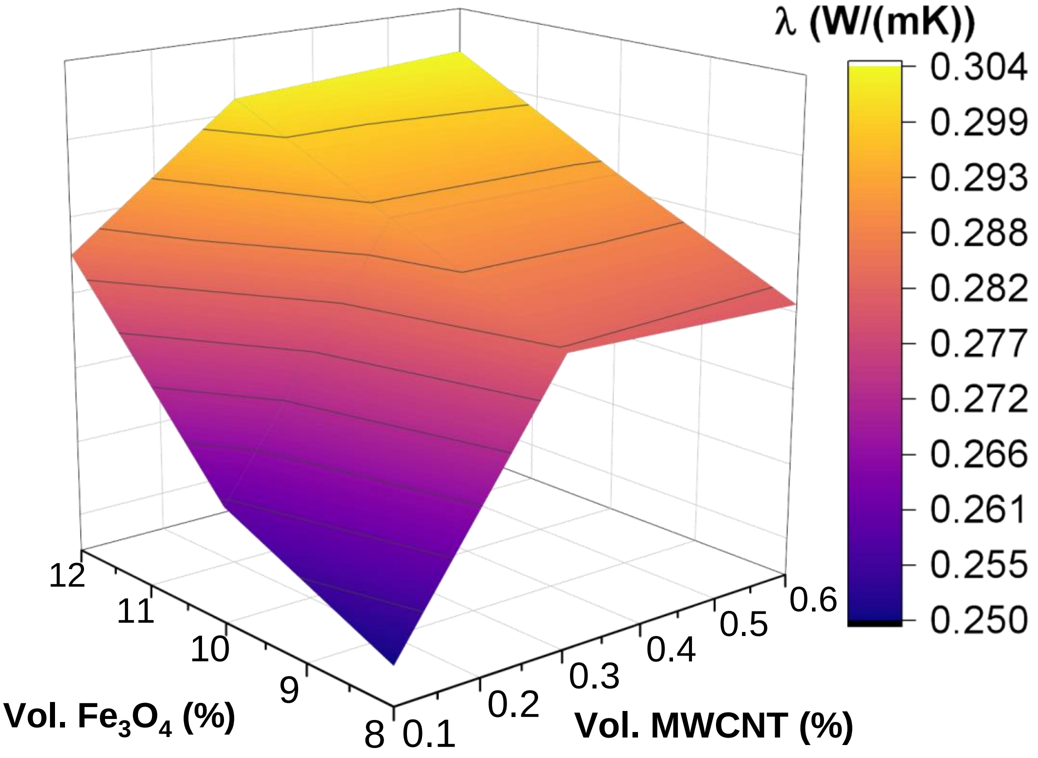

Figure S1. Surface plot of thermal conductivity of hybrid composites at 25 °C.

Table S1. Coded factors and initial Y values for the surface response model of Rs.

Composite	X_1	X_2	Y*
01-8	-1	-1	7.716
01-10	-1	0	7.677
01-12	-1	1	7.346
03-8	-0.2	-1	5.684
03-10	-0.2	0	5.552
03-12	-0.2	1	5.546
06-8	1	-1	4.081
06-10	1	0	4.126
06-12	1	1	3.758

* Decimal logarithm of experimental R_s.

Table S2. Calculated and adjusted regression coefficients for the response surface model of Rs.

Regression coefficient	Calculated	Adjusted
b_0	5.651	5.168
b_1	-2.309	-1.800
b_{11}	0.198	0.735
b_{12}	0.018	-0.018
b_2	-0.139	-0.192
b_{22}	-0.097	-0.150

Table S3. Parameters of the Arrhenius equation for PBS hybrid nanocomposites.

Sample	λ_0 (W/mK)	E_a (meV)
Ref	0.23	6.5
01-8	0.26	1.5
01-10	0.35	7.7
01-12	0.32	2.8
03-8	0.32	3.0
03-10	0.28	-1.4
03-12	0.32	3.0
06-8	0.38	7.4
06-10	0.38	7.0
06-12	0.40	6.7
